# Hemolytic disease of the fetus and newborn caused by anti-D and anti-S alloantibodies: a case report

**DOI:** 10.1186/1752-1947-6-71

**Published:** 2012-02-20

**Authors:** Rabeya Yousuf, Suria Abdul Aziz, Nurasyikin Yusof, Chooi-Fun Leong

**Affiliations:** 1 Blood Bank Unit, Department of Pathology, Universiti Kebangsaan Malaysia Medical Centre, Jalan Yaakob Latif, Cheras, 56000, Kuala Lumpur, Malaysia

## Abstract

**Introduction:**

Hemolytic disease of the fetus and newborn is most commonly caused by anti-D alloantibody. It is usually seen in Rhesus D (RhD)-negative mothers that have been previously sensitized. We report here a case of hemolytic disease of the fetus and newborn in a newborn baby caused by anti-D and anti-S alloantibodies, born to a mother who was RhD negative, but with no previous serological evidence of RhD alloimmunization.

**Case presentation:**

A one-day-old Chinese baby boy was born to a mother who was group A RhD negative. The baby was jaundiced with hyperbilirubinemia, but with no evidence of infection. His blood group was group A RhD positive, his direct Coombs' test result was positive and red cell elution studies demonstrated the presence of anti-D and anti-S alloantibodies. Investigations performed on the maternal blood during the 22 weeks of gestation showed the presence of anti-S antibodies only. Repeat investigations performed post-natally showed the presence of similar antibodies as in the newborn and an anti-D titer of 1:32 (0.25 IU/mL), which was significant. A diagnosis of hemolytic disease of the fetus and newborn secondary to anti-D and anti-S was made. The baby was treated with phototherapy and close monitoring. He was discharged well after five days of phototherapy.

**Conclusions:**

This case illustrates the possibility of an anamnestic response of allo-anti-D from previous sensitization in a RhD-negative mother, or the development of anti-D in mid-trimester. Thus, it highlights the importance of thorough antenatal ABO, RhD blood grouping and antibody screening, and if necessary, antibody identification and regular monitoring of antibody screening and antibody levels for prevention or early detection of hemolytic disease of the fetus and newborn, especially in cases of mothers with clinically significant red cell alloantibody.

## Introduction

Hemolytic disease of the fetus and newborn (HDFN) is characterized by the presence of IgG antibodies in the maternal circulation, directed against a paternally derived antigen present in the fetal/neonatal red cells that cause hemolysis in the fetus by crossing the placenta and sensitizing red cells for destruction by the macrophages in the fetal spleen [1]. It was first described in 1609 by a French midwife [2] but established in 1939 by Levine and Stetson. They reported a transfusion reaction from transfusing the husband's blood to a postpartum woman who had been immunized through a feto-maternal hemorrhage [3]. The serological tests for the diagnosis of HDFN includes a positive direct Coombs' test (DCT) on the baby's red blood cells and the presence of an IgG red cell alloantibody in both cord blood eluate and maternal sera. The presence of the corresponding antigen on cord cells confirms the diagnosis of HDFN [4,5]. The most severe HDFN is caused by IgG antibodies directed against D, c or K antigens on the fetal red cells, but any IgG antibodies can cause HDFN [6]. Anti-S has been documented as a rare cause of HDFN [7].

In this study, we report a case of HDFN caused by anti-D and anti-S in a para 3+1 mother who had no anti-D antibodies detected during the first trimester of pregnancy. The presence of allo-anti-D in the newborn and the mother herself postpartum may suggest an anamnestic response from previous sensitization or the development of anti-D during early trimesters of pregnancy. It also highlights the importance of regular monitoring of antibody screening in pregnant women, especially Rhesus D (RhD)-negative mothers, in view of the high immunogenicity of the RhD antigen.

## Case presentation

A full-term, Chinese baby boy was born to a 30-year-old woman at 38 weeks of gestation. The baby weighed 2.8 kg with an Apgar score of 9/10. The baby was noted to have mild jaundice with normal vital signs on day one of life; there was no evidence to suggest other causes of neonatal jaundice such as intrauterine infections and his glucose-6-phosphate dehydrogenase screen was normal. Laboratory investigations showed that his total bilirubin was 198 μmol/L and hemoglobin was 19 g/dL. The baby's blood group was A RhD positive with a red cell phenotype of ccDEe (R2r) and SS. The result of a DCT was positive and red cell elution studies of the baby's blood identified the presence of anti-D and anti-S antibodies.

The mother was para 3+1. Her first pregnancy was aborted five years ago and unfortunately no investigation was performed to find out the cause of abortion. Subsequent pregnancies were uneventful with no history of HDFN in the last three years. She denied any previous history of blood transfusion. Her transfusion record at our center showed that the mother developed anti-S antibodies during her second pregnancy three years ago. An antenatal antibody screening test performed at 22 weeks identified only allo-anti-S; no anti-D was detected. She was given RhD Ig prophylaxis at 28 weeks of pregnancy. Her other laboratory investigation results showed that she was grouped as A RhD negative with red cell phenotype ccdee (rr), and homozygous ss. At postpartum, the result of her DCT was negative, but the antibody screening test performed using the indirect Coombs' test method and antibody investigations showed the presence of anti-D and anti-S, and the anti-D titer was 1:32 (0.25 IU/mL).

In view of the presence of allo-anti-D and allo-anti-S in the postpartum maternal blood, supported by the presence of similar alloantibodies in the baby's red cell eluates and clinical presentation of hemolytic anemia, a diagnosis of hemolytic disease of the fetus and newborn secondary to anti-D and anti-S was made. The baby was immediately started on a course of conventional phototherapy with a single tungsten halogen bulb. His serum bilirubin levels subsequently reduced to normal levels over a few days. On the sixth day of life, the baby was discharged well with no complications.

## Discussion

This case illustrates an uncommon case of HDFN caused by anti-D and anti-S antibodies, which were identified from the red cell eluate of the baby as well as the mother's serum post-natally. In this case, there was no anti-D detected at 22 weeks of gestation and there was no subsequent follow-up at our center. However, at postpartum, when the newborn developed jaundice an investigation for HDFN demonstrated that there were both anti-D and anti-S. The possible explanation for the anti-D at postpartum could be due to (a) RhD Ig prophylaxis given at the early third trimester of pregnancy. A previous case report by Hensley *et al. *[8] showed that the maternal antibody screen becomes normal at 37 weeks of pregnancy in a mother who was given an RhD Ig prophylaxis at 28 weeks of pregnancy. The maternal serum sample was taken 40 minutes after RhD Ig immunization and showed an anti-D titer of 1:8, which subsequently peaked at a titer of 1:32 at 24 hours and remained at that level for about two weeks and then leveled off at 1:16 from week three through to week nine. At term, about 37 weeks of gestation, the maternal antibody screens reverted to normal [8]. Besides that, it is thought that the administration of RhD Ig during pregnancy can cause a positive antibody screening in the mother but the anti-D titer rarely reaches above 1:4 [9]. In our case study, the mother claimed that she was only given RhD Ig at 28 weeks of gestation and her anti-D titer was 1:32 (0.25 IU/mL). The high anti-D titer of 1:32 in our case study is most probably due to RhD alloimmunization from exposure of the mother to RhD positive fetal red blood cells later in gestation and unlikely to be due to the administration of RhD Ig at 28 weeks.

Another possible explanation could be (b) an anamnestic response to anti-D. The mother could have had previous exposure to the RhD antigen during her previous abortion or pregnancies, and these anti-D antibodies were not initially detectable in her plasma but subsequent exposure to the RhD antigen from this pregnancy at a later point provoked a rapid and robust production of anti-D antibodies. The titer detected was also significant, as it is described in the literature that anti-D titers of ≥1:32 are significant and can lead to HDFN [3,9]. Unfortunately, we were unable to differentiate between the two concentrations without the regular monitoring of antibody screening and identification and quantification of the antibody titer in our patient.

Anti-S antibody is an IgG antibody developed following red cell alloimmunization. It is reactive at 37°C and best detected by the indirect antiglobulin test. A literature search revealed that anti-S is a rare cause of HDFN and usually presents as a mild form of jaundice [7]. However, there are a few reports of severe and fatal HDFN due to this antibody. The first case of severe HDFN due to anti-S was described as early as 1952 where a baby died secondary to kernicterus at 60 hours of life [10]. Griffith [11] and Mayne *et al. *[12] also identified two other cases of severe HDFN due to anti-S. Fortunately, in our patient, despite the presence of anti-D and anti-S on the baby's red cells, the severity of HDFN was relatively mild and the baby's condition improved with phototherapy.

## Conclusions

In this report of an uncommon case of HDFN due to anti-D and anti-S antibodies, the detection of anti-D in postpartum serum could be explained by the anamnestic effect of previous alloimmunization or the sensitization after 22 weeks of gestation in the mother's current pregnancy. This case report highlights the importance of regular antenatal follow-up and monitoring of red cell alloantibody development and antibody titers, especially in a mother who is RhD negative. This is because the red blood cell alloantibody may lead to HDFN of variable severity, and early detection *in utero *may allow early intervention and thus minimize morbidity at birth.

## Consent

Written informed consent was obtained from the patient's legal guardian for publication of this case report and any accompanying images. A copy of the written consent is available for review by the Editor-in-Chief of this journal.

## Competing interests

The authors declare that they have no competing interests.

## Authors' contributions

RY obtained the case history and consent from our patient's mother. SAA and NY analyzed and interpreted our patient's laboratory investigation results and assisted with the literature review. RY and LCF played a major role in the literature review and writing the manuscript. LCF edited the manuscript. All authors read and approved the final manuscript.
